# Barriers to Accessing Treatment Services: Child Victims of Youths with Problematic Sexual Behavior

**DOI:** 10.3390/ijerph18105302

**Published:** 2021-05-17

**Authors:** Alexandra Slemaker, Peter Mundey, Erin K. Taylor, Lana O. Beasley, Jane F. Silovsky

**Affiliations:** 1 Department of Sociology and Criminal Justice, Iowa State University, 510 Farm House Lane, Ames, IA 50010, USA; slemaker@iastate.edu; 2 Department of Social and Behavioral Sciences, Savannah State University, 3219 College Street, Savannah, GA 31404, USA; mundeyp@savannahstate.edu; 3 Department of Pediatrics, University of Oklahoma Health Sciences Center, Oklahoma City, OK 73104, USA; erin-taylor@ouhsc.edu; 4 Department of Human Development and Family Science, Oklahoma State University, 338 Human Sciences, Stillwater, OK 74078, USA; lana.beasley@okstate.edu

**Keywords:** problematic sexual behavior, sibling incest, child sexual abuse, sibling sexual abuse (SSA), evidence-based treatment, child-on-child sexual abuse (COCSA)

## Abstract

Child sexual abuse (CSA) remains a significant public health problem. Although the deleterious effects on the child victims could be mitigated through evidence-based interventions, victims often fail to be identified and receive clinical assessment and therapy services, particularly when they have been victimized by another youth. Given that at least a third of CSA cases are committed by another youth, understanding the process of identifying and addressing the needs of CSA victims of youth is the focus of the present study. Factors impacting services for child victims of youths with problematic sexual behavior (PSB) were examined through qualitative interviews (N = 226) with mental health agency administrators, direct service providers, and community stakeholders from eight geographically diverse communities across the United States. Responses focused on macro and micro level barriers to the identification and service provision for child victims of PSB of youths. Implications for clinicians and policymakers are discussed, along with strategies to enhance access and provision of services to meet the needs of the child victims.

## 1. Introduction

Childhood sexual abuse (CSA) has deleterious effects that make it a significant public health problem worldwide [1,2,3,4,5]. In a meta-analysis by Stoltenborgh and colleagues [5], the lowest rates of CSA were found in Asia, while Australia has the highest rates of CSA for girls and Africa had the highest rates for boys. Although official rates of CSA in the United States (U.S.) are declining [6,7,8], it remains a significant public health problem [2,4,9]. The impact of CSA is wide ranging, with notable deleterious emotional, social, behavioral, and mental health outcomes for victims [10,11,12,13,14,15]. Immediate impacts of depression, anxiety, dysthymia, and other mental health concerns may manifest [16]. Due to CSA leading to an increased likelihood of re-victimization, these adverse outcomes compound [17,18,19,20].

The impact of CSA is multilayered, with outcomes on the individual level such as mental health, job productivity, health, and well-being, as well as on social and service systems level (i.e., legal, social services, mental health) [10,11,12,13,14,15,21,22]. As a result, estimates of the itemized economic costs of CSA are sizeable [12,23,24]. For instance, in 2015 alone, the lifetime cost of CSA was estimated to be $9.3 billion in the U.S., which included victims’ health care costs, productivity losses for caregivers, and costs to child welfare systems [25].

Although most of the extant CSA literature focuses on cases in which an adult abused the child, interpersonal CSA among youths has gained increased attention in recent years. Interpersonal problematic sexual behavior (PSB) of youths toward other children can be a form of CSA [26,27]. The term PSB encompasses a wider set of sexual behaviors that are developmentally inappropriate and potentially harmful to the child themselves or others [26]. Interpersonal PSB reflects acts among more than one child and can be considered a form of CSA when the behavior harms or has the potential to harm the other children.

### 1.1. CSA: Interpersonal PSB among Youths

When examining the wider issue of CSA committed by youths, it has been estimated that over one-third of CSA cases in the U.S. and United Kingdom are committed by other youths (e.g., neighbors, extended family, peers) [28,29]. However, this figure is likely an underestimate of the true prevalence of CSA committed by another youth, as victims may not report the abuse for a variety of reasons (e.g., they may anticipate a lack of support or anger from their caregivers if they report the incident) [30]. When examining youth reports rather than official records, rates of sexual abuse by another youth (rather than an adult) rise to approximately 75% [31]. Thus, many cases of CSA among youths appear to be unreported and not identified by professionals.

Youths with PSB exhibit significant heterogeneity in their demographic characteristics, causal factors, and socio-ecological features. PSB can begin at ages as young as 3 or 4 years old (Silovsky & Niec, 2002). Notably, the relationship between gender and PSB changes over time. Females constitute a greater portion of youths with PSB in early childhood years and preteen years, but males become more likely to exhibit PSB during adolescence [28]. Male children were equally likely to be abused by youths with PSB, while female children are more likely to be abused by male youths with PSB [31]. However, further research on child victims of PSB is largely limited.

Research on youths with interpersonal PSB has examined the relationship types of the children involved. Rather than strangers, victims of youths with PSB are commonly known to the youths, such as school mates, acquaintances, and family members, particularly siblings [28,32,33,34,35,36,37]. There is a growing body of literature focused specifically on sibling sexual abuse (SSA), and it represents an important sub-category of the CSA literature [33,34,35,36,37,38]. Some research suggests SSA occurs more often than caregiver-perpetrated CSA [35,39,40,41,42], although SSA occurs within only a minority of families. In 2014, approximately 2.3% of children in the U.S. were found to be victims of SSA [35,42].

Emerging research suggests that CSA victims of youths may experience similar psychological and health detriments as those victimized by adults [43,44,45], with wide ranging reactions including confusion, imitative behaviors, anxiety, behavioral disruptions, and trauma symptoms. Thus, research on identifying CSA victims of youths, linking to clinical assessments, and triaging to targeted effective interventions is needed to develop strategies to mitigate the negative effects of CSA.

### 1.2. Facilitators & Barriers for Treating Victims of CSA

Significant headway has been made in the availability and efficacy of behavioral health treatments for victims of CSA. In 1984, the U.S. Congress passed the Victims of Crime Act (VOCA), which established a fund that has provided millions of dollars to states across the U.S. for the treatment of victims of any crime, including CSA (https://www.rainn.org/articles/victims-crime-act) (accessed on 3 March 2021). Moreover, highly effective evidence-based treatments for child victims of CSA have been developed. For example, Trauma-Focused Cognitive Behavior Therapy (TF-CBT) is a short-term (16–20 sessions), structured, outpatient treatment designed to address posttraumatic stress symptoms caused by trauma and maltreatment [46]. The evidence supporting TF-CBT for trauma effects on youth victims of sexual abuse is quite strong with multiple randomized clinical trials demonstrated a positive impact on a range of behavioral health issues [15,47,48].

Unfortunately, efficacious treatments are often not provided to victims of CSA due to challenges at the individual, family, agency, service system, and policy levels. More broadly, overall CSA victims may not disclose their abuse, which would mean that they would not receive appropriate, trauma-informed services [30,49]. Additionally, child victims of CSA by another youth may not characterize their experiences as abusive [42]. In some contexts, abusive sexual behaviors between youths may be perceived as a normal part of youth culture rather than something to be disclosed, reported, or challenged [50]. Similarly, caregivers and institutional authorities may also be unable to accurately assess sexual behaviors to determine where they may fall on a continuum of healthy, typical, concerning, problematic, and harmful sexual activities in youth, thus repeatedly failing to recognize that abuse has occurred [36,51,52]. Finally, CSA among siblings is often minimized or ignored by caregivers who want to protect the privacy of their family, keep their home intact, and/or shield their child with PSB from being placed in the criminal justice system [34].

At the system level, misconceptions and stigma have led to ignored, resisted, or underfunded public health efforts to ameliorate CSA [2]. Rather than decisions based on the accumulation of evidence, policymakers may react emotionally when implementing legislation and public policies based on a single case with strategies not found to effectively prevent future CSA. Moreover, the criminal justice system in the U.S. is overtaxed, lacking the capacity, expertise, and infrastructure to support victims of CSA effectually [9].

Thus, a growing body of research is documenting the prevalence and potential impact of CSA committed by youths, with limited information on strategies of identifying cases and pathways to successful linkages of victims to evidence-based therapy. The current study is designed to fill that gap by examining the professional response to CSA committed by youths. As such, the goal of the present qualitative study is to elucidate how communities identify cases of CSA committed by youths and how they respond, including if and how they refer the child victims to effective interventions. Barriers (both concrete and perceptual) in identifying the child victims and the process of referring to and accessing services in communities across the U.S. are examined.

## 2. Materials and Methods

### 2.1. Study Design & Site Information

Data came from the Community-Based Services for Problematic Sexual Behavior of Youth Project (2014–2016), a mixed-methods research project studying eight communities that received funding from the Office of Juvenile Justice and Delinquency Prevention (OJJDP) to implement coordinated care for youths with PSB (ages 10 to 14) and victims from their intrafamilial (e.g., siblings, cousins) and social (e.g., friends, classmates, neighbors) networks. These communities reflect a mixture of large and medium-size metropolitan (i.e., urban, suburban; N = 7) and rural locations (N = 1) and are geographically diverse (i.e., West (N = 2), Midwest (N = 2), South (N = 1), and Northeast (N = 3)). Within each community, a services agency (e.g., children’s advocacy center, a children’s advocacy center (CAC) is a community-based, multidisciplinary, child friendly agency that provides services for children and their families who have been affected by abuse. These agencies are designed to create a safe place for children by coordinating the investigation of child maltreatment and response of law enforcement, child protective services, prosecutors, health professionals and others (https://www.nationalchildrensalliance.org/cac-model/) (accessed on 6 May 2021)., youth services, behavioral health) was selected to provide and coordinate services in conjunction with a multidisciplinary team (MDT). For the current project, we examined a subset of the qualitative data collected that focused exclusively on information regarding victim accessibility (or lack thereof) to services.

### 2.2. Participants & Interview Procedures

A purposive sample design was used to capture the perception of professionals in multiple communities across the U.S. Between July 2014 and August 2016, qualitative interviews were conducted with agency administrators (N = 51), direct service providers (N = 65), and community stakeholders (i.e., child welfare *n* = 17, juvenile justice *n* = 21, court *n* = 15; law enforcement *n* = 10; children’s advocacy center *n* = 18, mental health *n* = 22; and victim advocates *n* = 2; total N = 110). Interviewees were recruited by representatives from the lead service agency in each community. A total of 226 interviews were conducted with 137 unique individuals, several of whom were interviewed two or three times (see Table 1). Interviews occurred across three waves of data collection: Wave 1 (N = 84; July 2014–July 2015), Wave 2 (N = 73; June 2015–January 2016), and Wave 3 (N = 69; February 2016–August 2016), thereby allowing for analysis of victim services over a two-year period. Across all three waves of data collection, the research team was interviewed 226 of the 285 administrators, providers, and stakeholders who were identified by the lead service agency in each community and provided us with permission to contact them for an interview, resulting in a response rate of 79%. Participants were offered a USD 25 gift card to compensate them for their time.

Semi-structured qualitative interview guides were developed for each interviewee type (administrator, provider, or stakeholder), which contained questions and probes tailored to their specific role in the treatment of youths with PSB and their victims. Interview guides addressed a range of topics, with the current analysis focusing on responses relevant to questions about the child victims. Administrators and providers were asked about the services provided to victims and changes in these services over time. Administrators and stakeholders were interviewed about policy changes needed to improve support for child victims. Stakeholders were further asked about the process of accessing services for victims and their caregivers, while administrators were asked about current funding sources for victim services.

Phone interviews began with informed consent procedures, which were conducted by trained qualitative researchers, and lasted between 30–60 min. Interviews were digitally recorded and transcribed verbatim with the exception of de-identified data. Two transcripts were not able to be transcribed due to corrupt audio. To ensure quality control, all transcripts were cross-checked by another member of the research team. All research methods were approved by relevant Institutional Review Boards.

### 2.3. Qualitative Coding and Analysis

Interviews were systematically analyzed by a team of three qualitative researchers (the first three authors) using QSR N*Vivo 11 (QSR International, Chadstone, Victoria, Australia) to conduct keyword/term searches. For Wave 1 data, two researchers searched all transcripts for “trauma”, “abuse”, and “victim”. These searches also captured stemmed words (e.g., “victims” and “victimization”). Segments of text containing the keywords/terms were then coded into emergent themes/categories, including barriers to victim services. Barriers were not explicitly asked about in the interview, and instead emerged organically in the interviews. This emergent (as opposed to a priori) theme served as the basis for the Results presented below.

For the Wave 2 and Wave 3 interviews, one qualitative researcher applied the Wave 1 coding schema and methodology to code these data. Modifications were made to the coding schema as needed in order to account for new questions asked at the later waves, including questions about changes over time in victim services, and additional themes not previously found in the Wave 1 data. Upon coding all three waves of data, two members of the research team engaged in focused coding [53], which involved removing extraneous data, combining redundant themes, and creating overarching nodes that best synthesized the data from all three waves into the most analytically salient categories. Throughout all analyses, discrepancies were discussed among team members to ensure the reliability of coding.

## 3. Results

The major theme was how child victims often “fall through the cracks”, in the words of one of the stakeholders, such that, the child victims in the PSB cases were often overlooked in terms of the PSB case; the majority of the attention and services were focused on the youths with PSB. Qualitative interview data revealed barriers to identifying child victims and to referring and accessing evidence-based services. Interviewee-identified barriers to victims receiving effectual services were varied in nature, ranging from macro-level system issues (e.g., problematic policy & protocol, lack of service providers and long waitlists, ineffective inter-agency collaboration) to micro-level concerns (e.g., difficulty engaging and getting “buy in” from caregivers and victims). We detail these barriers in-depth below.

### 3.1. Problematic Policy & Protocol

A common policy-related concern reported revolved around cases in which no agency was designated as responsible to respond to youth-initiated CSA. For example, an administrator described a state law that prohibited investigations of interpersonal youth sexual abuse by child welfare because “[Child Welfare] does not have any statutory regulations or protocol to interview in that case because the youth on another youth did not have [guardianship of] the other youth. So, what happens right now is that often these cases that are reported are never even investigated.” Other interviewees reported that law enforcement did not have mandated protocols for investigating reports of youths with PSB and their victim(s), which often led to PSB cases being ignored. The reluctance to investigate or intervene often revolved around the youths with PSB being too young to be formally charged, which indirectly led to the minimization of the needs of the victim. Schools were also identified as not addressing reported incidents of youth-initiated CSA properly, as noted by the following stakeholder quote: “The kid is thinking ‘I told someone, I’ve told someone in an authority and nothing is ever been done about it.’ And so, it gets dropped and maybe the kid never told the parent and the school’s not telling the parent. We’ve had a couple of cases like that.”

Even if CSA is reported to the authorities designed to protect victims, state and federal policies about confidentiality often restricted the process of access to services by victims and their caregivers. Securing permission for MDTs to share information about the victims with the PSB treatment agencies was a significant challenge noted by interviewees, especially when the victim was not related to the youth with PSB. An administrator of a treatment agency designed to serve the youth with PSB and child victims observed,

We struggle with, because of HIPAA and confidentiality, of always identifying who the victims are. Sometimes we can if they’re cousins or extended family members, but we aren’t always able to access that information, especially if it’s a classmate or a neighbor child that we don’t have access to the families. We have lost [access to] several victims [because of this].

Similarly, some stakeholders identified struggles with permission to discuss victims and the youth with PSB at MDT meetings because of privacy laws, as seen in the following quotation: “You can’t openly discuss juveniles under any circumstances without having releases.” Treatment providers also had difficulty receiving victim referrals because other agencies (e.g., child welfare, juvenile justice) were prevented from releasing victim information due to confidentiality laws.

### 3.2. Struggles Promoting Education, Awareness & Acceptance

Interviewees described issues surrounding the lack of education and awareness about sexual victimization and its effect on the victim. Some interviewees discussed widespread underreporting of sexual victimization of youth by other youth, as seen in the following excerpt from a stakeholder interview: “We don’t see those numbers rise unless the kids report it and I’m sure many, many, many of these allegations and reports of sexual abuse go unreported.” The lack of education or understanding regarding the effects of sexual victimization on children among law enforcement was also discussed. “There’s like a million different ways that someone can be victimized,” a stakeholder stated in reference to how some police departments, especially those in rural areas, “don’t have the understanding or the awareness of the impact.” Other interviewees asserted that there was a systemic failure to grasp the severity of youth-initiated CSA or an avoidance of the issue altogether. According to a stakeholder:

Sometimes a law enforcement agency is asked by us to do a little extra work, especially in terms of getting siblings interviewed to rule them out as victims. And that’s really our issue with this one agency. They say they don’t have the time to do it. Of course, we’re trying to press the point that, “Hey this is a child in your community that may be a victim, and you’re not really interested in getting an answer to that question.”

Another stakeholder reported “a lot of friction” with a child welfare caseworker who did not want to address allegations of sexual abuse of youth by other youth:

We were doing an assessment, and the case had just been closed, and I had to hotline it based on facts that the kid disclosed to me in the assessment. And the caseworker called me the next day and was very angry and almost borderline kind of really attacking me, which I understood later she did not want this case to come back to her, but ya know, I am a mandated reporter.

In the absence of persistent victim advocates like this stakeholder, the tendency to minimize and conceal allegations of youth-on-youth sexual abuse could ultimately lead to victims being ignored by the system.

### 3.3. Ineffective Inter-Agency Communication and Collaboration

In addition to policy-related challenges, poor inter-agency communication and collaboration were notable impediments to victims receiving services and referrals. “That’s been the consistent problem from the beginning,” a provider stated regarding the lack of victim referrals they were receiving from their local CAC, adding, “that over the last four months, it’s gotten a little more difficult, you know, or they stop referrals to us altogether.” A different treatment provider similarly lamented how their CAC was “trying to implement their own rules about how they want us to take referrals.” Specifically, the CAC was asking the treatment agency to bypass their intake process and take clients without any initial evaluation, which the mental health provider noted was problematic because the intake is needed to guide treatment.

In other cases, relationships between provider agencies and law enforcement were reported to be strained. In a typical process, victims were to be brought to the CAC for a forensic interview in order to start the triaging process. Without this step, however, victims were not referred to treatment. An administrator, put it this way:

We’re having some issues with some of our law enforcement, our local law enforcement, in that they were not kind of abiding by the protocols of working within Child Advocacy Center model, and so now [they] are having a bit of a temper tantrum and not wanting to bring interviews to the Advocacy Center for our victims.

In still other cases, respondents indicated that communication among community partners (e.g., child welfare, schools, law enforcement) outside of the provider agency often broke down. “Our probation department and our [child welfare], they don’t talk to each other,” an administrator stated, “they don’t have systems, or computers, or really the vehicle where they even communicate. And since we’re huge in [our] county, we’ve always struggled with that.”

### 3.4. Difficulty Accessing or Receiving High-Quality Services

Even when sexual victimization is acknowledged, victims may have had difficulty accessing or receiving high-quality services. A few interviewees described waitlists for services or a lack of victim services providers in their area. One stakeholder said, “if we go outside the [agency], I think the services are more limited. There aren’t that many clinicians that are willing to work with sexual abuse victims or perpetrators.”

Even when victims located a therapist who was willing and able to provide services, additional barriers arose to accessing these services. In the words of one administrator, “some of the barriers are poverty and can they physically get to the appointments that they need to.” This was indicative of how transportation and financial barriers were identified causing caregiver stress and impeding regular attendance at treatment. Further, in the event that victims could successfully get to the treatment facility, the services offered may not have been enough to meet their needs. Interviewees stated that victims often had numerous challenges beyond being sexually abused, such as Attention Deficit/Hyperactivity Disorder, living with caregivers who had limited parenting skills, and experiencing, in the words of one interviewee, various other “environmental factors,” all of which required additional case management support. Unfortunately, the availability of multipronged services was limited.

### 3.5. Trouble with Victim & Caregiver Engagement

A range of factors, in addition to the effects of poverty, unreliable transportation, and other factors, were reported as hindering the engagement of the victims and their caregivers. “It’s totally up to the victim if they want to take advantage of the services,” a stakeholder reported. As gatekeepers to the victims, enlisting caregiver buy-in was noted as essential to getting victims the help they need, although this was not always possible. According to an administrator, “we have come across a couple of parents or guardians who don’t feel the victim is needing services, so we are trying to get out in front of that. You know, you can’t force it.” In cases of intrafamilial PSB, interviewees sometimes reported that caregivers wanted to minimize the victimization of one child in order to protect the child with PSB from being labeled a “perpetrator,” which led them to be unsupportive of treatment for either child.

### 3.6. Trouble with Victim & Caregiver Engagement

When CSA was identified and reported, multiple agencies may have been involved with the family. When this was not well-coordinated, the multiagency response could result in an overwhelming and emotionally stressful situation that hindered, rather than helped, the family’s healing. One stakeholder reported that by the time the victim got to their office, prior to the formation of a CAC in their community, it “could be [the] fourth or fifth time they’ve had to recount that story, and that’s just very unfortunate that someone has to continue, especially at a young age, has to keep telling that story over and over again.” Another stakeholder described how multiple system response can be more harmful than helpful:

When the child is victimized, they’re made to do all these different services to help them. Kids at that age, they don’t understand that it’s actually to help them. They see it as something bad happened to me and now I have to go here, I have to go there, I have to do this I have [various agencies involved] in my life. I have the cops in my life, and the child with the sexually offending behavior, kind of nothing happens if they’re not being prosecuted.

It was therefore possible that the victim may be caught up in the system, while there was a lack of response to the youth with PSB, who was then perceived as having no treatment or repercussions. This imbalance and uncoordinated response to the youth with PSB and child victims in communities has the potential to be insensitive to the needs of the child victim.

## 4. Discussion

To better understand identification and service responses to child victims of youths with PSB, qualitative interviews were conducted with service agency administrators, stakeholders, and providers over a 25-month period at eight sites across the U.S. The major theme that emerged was the barriers to identifying victims and their subsequent access to mental health services. Barriers were multifaceted at the macro and micro level of problematic policy and protocol, misinformed professionals, ineffective inter-agency communication and collaboration, difficulty with service accessibility or receipt, victim and caregiver engagement challenges, and victims overwhelmed by multiagency response.

These findings resonate with previous research indicating that community response to interpersonal PSB among youth often involves a large number of agencies that may not work efficiently and collaboratively together across cases [29,54,55,56,57]. Indeed, a myriad of community agencies (e.g., health care, law enforcement, mental health, social services) may become involved with families after disclosure [58,59]. Navigating this complex network of systems can be difficult for child victims of PSB, the youth with the interpersonal PSB, their caregivers, and potential mental health service providers. The complexity heightens when the youth with PSB and child victims are within the same family. In a study out of Australia, it was found that there was also a lack of consistency across all of the agencies involved with intrafamilial PSB cases, and the need for coordinated and regulated service provision for both the child victim and the youth with interpersonal PSB was identified [60]. Efforts to collaborate and streamline the flow of information among multiple parties are necessary if victims are to be properly served.

Given that interpersonal PSB most commonly occurs among children within the same social network, the response benefits from accounting for the risk and protective factors within the social context. Rather than a single trajectory of response, such as an immediate and prolonged separation and extensive therapy for all children involved, triage pathways may be best informed by considering child victim response, well-being, vulnerability, and wishes, as well as risk, needs, and responsivity of the youth with PSB, caregivers, and others impacted. Early access to evidence-based services, with ongoing assessment of progress, is needed but often difficult to obtain in many communities.

Multiagency collaboration can mitigate the ill-effects of what can be, in at least some cases, insensitive procedures used to respond to child maltreatment (e.g., repetitive interviews, invasive medical exams, family separation, intimidating justice system proceedings) [59,61]. CACs hold great promise in remedying many problematic responses, particularly by helping to coordinate identification, assessment, investigation, and response through collaboration among stakeholder agencies within an MDT. CACs are utilized as a hub where victims can be interviewed by trained personnel about their experiences, which serves as the official record for the case. Thus, child victims are not forced to relive the trauma and abuse by having to give multiple interviews to each agency involved [62,63]. Additionally, CACs are able to coordinate the care of victims of child maltreatment, the youth with PSB, and the caregivers by collaborating with community stakeholders (e.g., doctors, lawyers, mental health providers, law enforcement) to create and support successful implementation of treatment plans that best suits the interest and well-being of the victim [62]. Better leveraging the resources of CACs, as well as expanding their reach and presence, would enhance services for the child victims and the youth with PSB.

The National Children’s Alliance, the professional and accrediting board of CACs in the U.S., has responded to the issues of PSB of youth with concentrated efforts to improve education and collaborative response [64]. These changes were in response to leadership in CACs recognizing the prevalence of PSB of youth, which is approximately 25% of the CSA cases seen at CACs [62]. Moving from categorized responses to children as either a “victim” or an “offender,” best practices for CACs is to address the need of all the children involved. The context of the family, relationships, social structure, as well as risk and protective factors, inform responses that facilitate accountability, rehabilitation, and healing. Misinterpretation of policies of CACs designed to protect child victims led some CACs to disallow fully serving and addressing cases of PSB of youth. Polices established on this misunderstanding, such as refusing to allow any youth with PSB to enter a CAC at all, fail to account for the complexity of cases. Some youths with PSB may also have experienced victimization. Coordination of responses to cases would benefit from collaborative MDT responses. Further, in some cases, children remain or return to the home, and treatment plans integrate sibling therapy services. Thus, rules based on categorizing children as either victims or perpetrators may result in outcomes counter to the goals of CAC. Instead, the establishment of clear agency policies that support the physical and emotional safety of all children is best practice. Guidance on strategies to reach these goals while serving child victims and youth with PSB within the CAC is now available [64].

Even with this guidance, communities struggled to implement a coordinated response that is responsive and integrates an understanding of the impact of social networks. Policies or misinterpretations in the implementation of such policies, designed to protect privacy may inadvertently hinder collaborative care. When part of the equation is known (e.g., a youth has interpersonal PSB), streamlined and sensitive strategies to identify the other parties could facilitate assessment and services where needed. Respondents indicated current policies and practices are wrought with barriers to address all parties’ needs.

Gaps in the child protection systems were notable by the participants across the U.S., there appears to be a limited number of policies and procedures designed to identify and respond to cases of interpersonal PSB of youth [65,66]. Most of the policies related to CSA, such as mandatory reporting laws, are specifically geared toward caregiver-perpetrated abuse of minors. Reform of policies and procedures at the community, state, and federal levels would allow for the examination of responses specifically tailored to instances of interpersonal-PSB among youth. Some states in the U.S. (e.g., Missouri, Kansas) have begun a process of reforming their child protective service policies to screen in (rather than out) interpersonal PSB of youth, with subsequent investigation, assessment, and safety planning conducted as needed [67]. An initial spike in referrals of cases was notable in Missouri (https://abc17news.com/news/2016/02/08/child-on-child-sexual-abuse-bigger-problem-than-thought-in-missouri/) (accessed on 5 May 2021). In the UK, a report regarding treatment for youth with problematic sexual behavior revealed that these youth were being treated as juvenile or child sex offenders. Policy practices were changed which treated youth with problematic sexual behavior as children instead of criminals (https://yjlc.uk/report-warns-of-children-being-wrongly-treated-as-mini-sex-offenders/) (accessed on 6 May 2021). Further monitoring and investigation of the impact on identification, response, and outcomes could inform usefulness and application in other jurisdictions.

Reform efforts should be mindful of the inherent complexity of interpersonal PSB between youth, a topic that does not lend itself to a blanketed response [68]. The relationship of the victim and youth with PSB (e.g., siblings, cousins, other family relations) and living status prior to the PSB (e.g., living together, separate homes) have important implications for the process. For instance, the inclination to keep the family together, although well-intentioned, may not fully take into consideration the needs of some child victims [69]. Alternatively, research suggests that when it can be done safely and is in the best interest of all children, the best practice may be to keep the family unit together while reforming the bonds that were damaged [70]. Thus, a single protocol is not appropriate; rather, assessing the family members and context is critical for initial and ongoing safety planning.

Future research exploring the triage pathways that best support the well-being of the child victim and the youth with PSB, accounting for the complexities of interpersonal PSB among youth. Subsequent studies should also explore strategies among victim treatment providers and stakeholder agencies to overcome the barriers identified by our interviewees. Strategies to identify ways to ameliorate the underreporting of youth-initiated CSA and to gain a more accurate accounting of how prevalence will be an important target of investigation. It is only by understanding the full scope of this public health problem that scholars and practitioners will be able to best meet the needs of child victims. To that end, nationally representative and longitudinal survey data on youth with PSB and children who had been sexually abused would be highly beneficial for scholars.

Although the current study benefited from the in-depth nature of qualitative data, there may be limited generalizability, particularly to rural/frontier areas, special populations, and communities outside of the U.S. Moreover, it is important to reiterate that we did not explicitly ask participants about barriers. The emergent nature of these barriers can be viewed as a strength of our study, but future qualitative research should endeavor to ask barrier-specific questions to assess if additional themes emerge specifically.

## 5. Conclusions

Accurate identification, clinical assessment, and responsive interventions for child victims of CSA, including youth-initiated CSA, is imperative. The current study identified barriers that prevented child victims of youth with PSB from being identified, clinically assessed, and accessing evidence-based treatment services. Greater education and awareness of clinicians, treatment administrators, community stakeholders, and policy makers is a critical first step. Improved public policy and practice targeting enhanced coordination and collaboration among service agencies aimed at remedying these impediments is recommended to break down barriers and improve child and family well-being.

## Figures and Tables

**Table 1:** Interviewee type by state—wave 1–3 (July 2014–August 2016).

U.S. Region	Service Agency Providers	Service Agency Administrators *	Stakeholders	Total by Site
West	18	13	19	50
Midwest	16	18	41	75
Northeast	19	14	40	73
South	12	6	10	28
Total by Interviewee Type	65	51	110	226

Source: Community-based services for PSB of youth project, 2014–2016. *Note:* * Twenty of the administrator interviews were conducted with administrator–provider hybrids. Although we interviewed these individuals as administrators—which is how they are classified in this table—they also could be classified as providers for certain analyses.

## Data Availability

The data presented in this study are available on request from the corresponding author. The data are not publicly available due to confidentialty of the participants.
